# Cell-free hemoglobin is associated with microcirculatory perfusion disturbances and acute kidney injury in rats on extracorporeal membrane oxygenation

**DOI:** 10.1186/s12871-025-03251-3

**Published:** 2025-08-20

**Authors:** Carolien Volleman, Dionne P. C. Dubelaar, Philippa G. Phelp, Roselique Ibelings, Anita M. Tuip-de Boer, Chantal A. Polet, Walter M. van den Bergh, Alexander P. J. Vlaar, Charissa E. van den Brom

**Affiliations:** 1 Department of Intensive Care Medicine, Amsterdam UMC, University of Amsterdam, Amsterdam, the Netherlands; 2 Laboratory for Experimental Intensive Care and Anesthesiology (LEICA), Amsterdam UMC, University of Amsterdam, Amsterdam, the Netherlands; 3 Department of Anesthesiology, Amsterdam UMC, VU University, Amsterdam, the Netherlands; 4 Department of Critical Care, University Medical Center Groningen, University of Groningen, Groningen, The Netherlands

**Keywords:** ECMO, Extracorporeal membrane oxygenation, Extracorporeal circulation, Endothelium, Edema, Hemolysis, Kidney injury, Microcirculatory perfusion, Cell-free hemoglobin

## Abstract

**Background:**

Extracorporeal membrane oxygenation (ECMO) is a life-saving treatment, but carries a high risk of complications such as acute kidney injury (AKI). A contributor to AKI is hemolysis, which induces vasoconstriction and renal tubular cytotoxicity. Here, we have investigated a novel hypothesis that ECMO-induced hemolysis contributes to vascular leakage, edema, microcirculatory perfusion disturbances, and AKI in a rat model.

**Methods:**

Rats were exposed to 75 min of ECMO or a sham procedure as control (*n* = 8 per group). Hemodynamic, blood gas, and microcirculatory perfusion parameters were monitored throughout the experiment. Renal vascular leakage and edema were determined by dextran leakage (70 kDa) and wet-to-dry weight ratio. Markers of hemolysis, inflammation, endothelial activation and damage, and AKI were assessed using spectrophotometry, ELISA and Luminex.

**Results:**

Initiation of ECMO increased circulating cell-free hemoglobin (CFHb) compared to baseline (4.01 vs. 1.36 OD, *p* < 0.001). In parallel, ECMO increased circulating levels of TNFα, IL-6, ICAM-1 and angiopoietin-2, whereas levels in the control group remained stable. The number of continuously perfused vessels (4.36 vs. 13.62 vessels/recording, *p* < 0.001) and the proportion of perfused vessels (PPV; 23.0 vs. 67.4%, *p* < 0.001) immediately decreased after initiation of ECMO when compared to controls and remained disturbed one hour after weaning from ECMO. Furthermore, NGAL, a marker of kidney injury, in plasma and urine was higher in the ECMO group compared to the controls (respectively 2191 vs. 410 ng/mL, *p* < 0.001; 1733 vs. 437 ng/mL, *p* = 0.0059). Wet-to-dry weight ratio showed increased renal edema in the group undergoing ECMO (4.50 ± 0.27 vs. 3.96 ± 0.16, *p* < 0.001). Moreover, increasing levels of CFHb in plasma were correlated with a decrease in PPV (*r*=-0.925, *p* < 0.001) as well as an increase in plasma NGAL (*r* = 0.895, *p* < 0.001) in rats on ECMO.

**Conclusion:**

In conclusion, ECMO-induced hemolysis is paralleled by endothelial damage, microcirculatory perfusion disturbances, and kidney injury in a rat model. Our findings suggest that CFHb plays an important role in the pathophysiology of AKI, possibly via endothelial damage. Future studies should clarify the causal relationship between CFHb and endothelial damage, and explore whether targeting CFHb can improve microvascular perfusion and preserve kidney function during ECMO support.

## Background

Extracorporeal membrane oxygenation (ECMO) provides support in case of potentially reversible cardiac and/or respiratory failure, refractory to conventional therapies. Despite technological advancements, morbidity and mortality remain high, with overall survival rates around 50% [1]. Among the most frequent complications is acute kidney injury (AKI), affecting almost two-thirds of patients, of whom three-quarters require renal replacement therapy [2, 3]. Nonetheless, knowledge of the pathophysiological mechanisms underlying AKI in patients on ECMO support is scarce, and effective preventive therapies are lacking.

During ECMO, mechanical trauma inflicted on red blood cells within the circuit can cause hemolysis, which currently affects 15–20% of patients receiving ECMO support [2, 4]. Intravascular hemolysis is characterized by an increase in cell-free hemoglobin (CFHb) due to rupture of the erythrocyte membranes. Under physiological conditions, CFHb is scavenged by haptoglobin, effectively mitigating its toxic effects. However, during pathophysiological conditions, haptoglobin can become depleted resulting in elevated levels of CFHb. These elevated CFHb levels are associated with AKI and increased mortality in patients supported by ECMO [5, 6], which highlights the need to understand how CFHb drives these adverse outcomes.

When freely circulating, CFHb exerts harmful effects in the circulation through three proposed mechanisms. Firstly, CFHb causes increased vasoconstriction due to decreased bioavailability of nitric oxide [7, 8]. Secondly, it leads to abnormal coagulation and platelet activation, promoting a prothrombotic state [9]. Lastly, CFHb induces renal tubular cytotoxicity as unbound CFHb is filtered by the glomerulus and is taken up by proximal tubular cells [10, 11]. However, these mechanisms do not fully explain the extent of renal injury observed in patients on ECMO support. Therefore, we propose a new hypothesis for ECMO-induced hemolysis and AKI.

ECMO initiates multiple pathological pathways, such as systemic inflammation and coagulation disturbances due to, amongst others, exposure of blood to foreign surfaces of the extracorporeal circuit [12]. These processes can activate the endothelium and may increase endothelial permeability leading to leakage of fluid to the interstitium, ensuing tissue edema and organ injury [12, 13]. Previously, we have demonstrated that extracorporeal circulation in rats increased endothelial permeability, which was paralleled by impaired microcirculatory perfusion and renal injury [14–17]. Moreover, pharmacological protection of the endothelium reduced vascular leakage and tissue edema and improved microcirculatory perfusion in rats undergoing extracorporeal support, emphasizing the endothelium’s importance in protecting organ function [14, 16, 17]. Novel interventions protecting the endothelium during ECMO support are therefore of high interest.

Recent in vitro studies suggest that CFHb directly activates endothelial cells, inducing inflammatory cytokine release and upregulation of adhesion molecules [18, 19]. While these findings in pulmonary endothelial cells suggest a critical role for CFHb in endothelial damage, it remains unclear whether similar mechanisms contribute to AKI. Therefore, this study aimed to elucidate the relationship between ECMO-induced hemolysis, microcirculatory perfusion disturbances, and AKI. We hypothesize that ECMO-induced hemolysis, as represented by increased CFHb, contributes to microcirculatory perfusion disturbances and AKI, potentially through endothelial damage.

## Methods

### Animals and experimental set-up

All procedures were approved by the Institutional Animal Care and Use Committee of the University of Amsterdam, the Netherlands (Animal welfare number: AVD1140020172144), and conducted following the EU Directive (2010/63EU) on the protection of vertebrate animals used for experimental and other scientific purposes and the ARRIVE guidelines on animal research [20].

Male Wistar rats weighing 375–425 g (Charles River Laboratories, Brussels, Belgium) were housed in a temperature-controlled room (12/12 h light-dark cycle, 20–23 °C, 40–60% humidity) with food and water *ad libitum*.

**Fig. 1:**
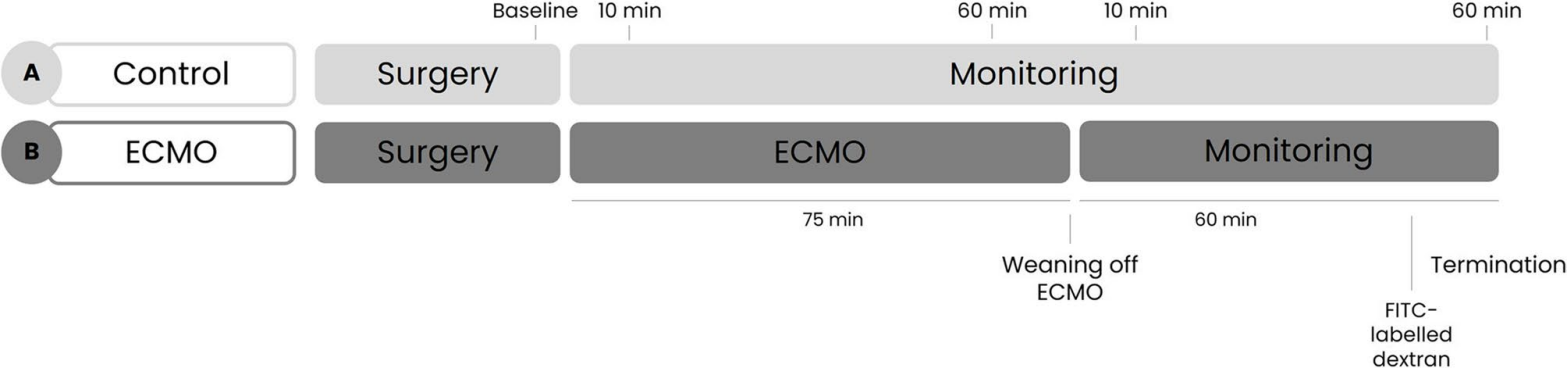
Experimental set-up. Rats were randomly assigned to undergo 75 min of extracorporeal circulation (**B**) or a sham procedure as control (**A**; *n* = 8 per group). After weaning from ECMO, the rats were monitored for one hour after which they were terminated. Prior to termination, FITC-labeled dextrans were injected to determine vascular leakage. Hemodynamic measurements, microcirculatory perfusion measurements, and blood withdrawal for blood gas analyses were performed at baseline, 10 and 60 min after initiation of ECMO and 10 and 60 min after weaning from ECMO. Plasma, urine and kidneys were collected and stored for analyses

Rats were randomly assigned to undergo 75 min of ECMO or a sham procedure as control (*n* = 8 per group). After discontinuation of ECMO, the rats were monitored for one hour after which they were terminated by exsanguination under 5% isoflurane inhalation (Fig. 1). The rats in the control group underwent identical surgical preparation, but were not connected to ECMO. Instead, they were monitored consecutively for 135 min. Hemodynamic and microcirculatory perfusion measurements and blood gas analyses were performed at baseline, 10 and 60 min after initiation of ECMO, and 10 and 60 min after weaning from ECMO. At the same time points, blood was withdrawn for plasma storage. After termination, urine and kidneys were isolated and stored for analyses.

### Anesthesia and surgical preparation

All animals were anesthetized using a mixture of 4.0% isoflurane (Karizoo, Barcelona, Spain) and oxygen-enriched air, followed by endotracheal intubation with a 14G catheter (Venflon Pro, Becton Dickinson, Helsingborg, Sweden) [16, 17, 21]. The lungs of the rats were mechanically ventilated (UMV-03, UNO Roestvaststaal BV, Zevenaar, The Netherlands; PEEP 2–4 cm H_2_O, respiratory rate of 60–80 breaths/min, tidal volume ~ 10 ml/kg) and anesthetics were maintained at 1.0–2.0% isoflurane in oxygen-enriched air (40% O_2_, 60% N_2_). Ventilatory settings were adjusted based on blood gas values to maintain pH and partial pressure of carbon dioxide within physiological limits (pH 7.35–7.45; pCO_2_ 35–45 mmHg). Depth of anesthesia was adjusted based on heart rate and mean arterial pressure. Fentanyl boluses (12 µg/kg, Janssen-Cilag, Tilburg, the Netherlands) were given approximately every 45 min as additional analgesia. The carotid artery was cannulated using a polythene cannula (ICU Medical, Houten, Netherlands) for continuous arterial blood pressure measurements and blood withdrawal for blood gas analyses and hematocrit measurements (RAPIDPoint 500, Siemens, Munich, Germany). Arterial blood pressure, ECG, and heart rate were continuously recorded using Powerlab software (PowerLab 8/35, Chart 8.0; AD Instruments Pty, Ltd., Castle Hill, Australia).

The left cremaster muscle was isolated under warm saline superfusion, spread out on a heated platform (34 °C), and covered with gas impermeable plastic film (Saran wrap). Cremaster perfusion measurements were performed as previously described [16, 17, 21, 22].

The right jugular vein was cannulated for venous inflow of the ECMO circuit using a modified PerkuFlow n. Schlottman (Pflugbeil GmbH, Zorneding, Germany). For arterial flow from the circuit back to the rat, the right femoral artery was cannulated with a 20G catheter (Arterial Cannula, Becton Dickinson, Helsingborg, Sweden). All catheter insertions were preceded by local application of 1% lidocaine. Heparin (500 IU/kg, LEOPharma, Amsterdam, the Netherlands) was administered prior to canulation of the right jugular vein. An additional dose of heparin (500 IU/kg) was given in combination with rocuronium bromide (1.5 mg/kg, Fresenius Kabi, Halden, Norway) before initiation of ECMO. The sham animals underwent identical surgical procedures, including intubation, mechanical ventilation, cannulations and cremaster isolation. Additionally, they did receive equivalent doses of heparin and rocuronium. However, the sham animals were not connected to the ECMO circuit but were monitored for 135 min following surgery.

### Extracorporeal circulation protocol

The ECMO circuit consists of an open venous reservoir, a roller pump (pericor SF70, Verder, Haan, Germany), and an oxygenator-heat exchanger consisting of a three-layer hollow fiber membrane for gas exchange (Ing. M. Humbs, Valley, Germany). The catheter in the femoral artery was connected to a 1.0-mm-diameter arterial line (LectroCath, Vygon, Ecouen, France). Target ECMO flow rates (> 140 ml/kg/min) were maintained, and additional doses of albumin were administered when necessary. Blood flow rates in the ECMO circuit were measured using a small animal blood flow meter (T206 Transonic Systems, Ithaca, NY, USA). To maintain mean arterial pressure (MAP) ≥ 50 mmHg, boluses of phenylephrine (10 µg) were administered to sustain organ perfusion pressure. After 75 min, the rats were weaned from ECMO by removing the venous cannula and clamping the jugular vein.

### Microcirculatory perfusion measurements

The exposed cremaster muscle was left to stabilize for at least 30 min before the first microcirculatory perfusion measurements were performed. An intravital microscope (AxiotechVario 100HD, Zeiss, Oberkochen, Germany) with a 10x objective was connected to a digital camera (acA720-520uc, Basler, Ahrensburg, Germany) with a final magnification of 640x, as described previously [16, 17, 21, 22]. Three regions of adequate quality were selected (vessels up to 20 μm diameter) for baseline and all subsequent measurements (Fig. 1).

Analyses of microcirculatory perfusion were performed offline by counting capillary crossings with two vertical test lines in each video screen. These small vessels were scored as continuously perfused, intermittently perfused (blood flow was arrested or reversed at least once), or non-perfused (vessels without erythrocytes or non-flowing erythrocytes). The total vessel density was defined as the total number of small vessels of all three categories. The proportion of continuously perfused vessels (PPV) was calculated by the ratio of the absolute number of continuously perfused vessels (PVD) and the total vessel density. Two investigators independently scored all videos and were blinded to the allocated experimental group.

### Plasma protein analyses

Arterial blood was collected at set time points as shown in Fig. 1. Urine samples were collected after termination. Levels of interleukin-6 (IL-6), tumor necrosis factor α (TNFα) and intercellular adhesion molecule 1 (ICAM-1) were measured using a Luminex platform (Biotechne, Minneapolis, MN, USA). Circulating levels of angiopoietin-2 (SEA009Ra, Cloud-Clone Corporation, Katy, TX, USA), haptoglobin (NBP2-60528, Biotechne, Minneapolis, MN, USA), and neutrophil gelatinase-associated lipocalin (NGAL; ab119602, Abcam, Cambridge, UK) were measured with enzyme-linked immunosorbent assay (ELISA) in accordance to the manufacturer. To account for changes in the plasma compartment, all plasma marker concentrations were adjusted for hematocrit level fluctuations relative to baseline. This correction was applied by dividing the measured plasma concentration per unit plasma volume by the hemoconcentration factor h(p) calculated as h(p) = H(x) [100 − H(b)]/(H(b) [100 − H(x)]), where H(b) represents the baseline hematocrit (in percent) and H(x) the hematocrit at any given time point x.

### Lactate dehydrogenase

Lactate dehydrogenase (LDH) activity was analyzed by spectrophotometry (Spectramax M2e, Molecular Devices, San Jose, CA, USA) at 340 nm at 25 °C. 0.5 M potassium phosphate buffer and 10% Triton were added to our sample. Sodium pyruvate was added as substrate, after which the rate of NADH oxidation was measured [23]. The decrease in NADH concentration reflects the total LDH activity.

### Cell-free hemoglobin

CFHb was determined using Drabkin’s method [24]. Plasma and urine samples were diluted in Drabkin’s reagent (D5941, Sigma Aldrich, Saint Louis, MO, USA) and incubated for 15 min at room temperature. The absorbance of the samples was measured at 540 nm using spectrophotometry (Spectramax M2e, Molecular Devices, San Jose, CA, USA).

### Renal edema

Kidney tissue was harvested at the end of the experiment after termination of the animal. Wet tissue of the left kidney was weighed and dried at 37 °C and weighed again after 48 h. Wet-to-dry weight (W/D) ratio was calculated to estimate tissue water content.

### Renal vascular leakage assessment

Vascular leakage was determined by extravasation of FITC-labeled dextrans as previously described [25]. FITC-labeled dextrans (6.25 mg, 70 kDa; FD70S-1G, Sigma-Aldrich, Saint Louis, MO, USA) were administered intravenously fifteen minutes prior to termination. After termination, the animal was systemically flushed with saline, apart from the left kidney which was ligated to preserve for W/D ratio. Tissue of the right kidney (50 mg) was homogenized in RIPA buffer with protease and phosphatase inhibitors (cOmplete^™^ Protein Inhibitor Cocktail, Roche) and centrifuged for 15 min at 13,000 *g* at 4 °C. Fluorescence intensity of kidney homogenates was determined at an excitation wavelength of 485 nm and an emission wavelength of 528 nm using spectrophotometry (Spectramax M2e, Molecular Devices, San Jose, CA, USA) and converted to µg/mg tissue using a standard curve.

### Statistical analysis

Sample size was calculated based on intraoperative cell-free hemoglobin concentrations in patients undergoing cardiac surgery with or without cardiopulmonary bypass (13 ± 6.6 vs. 2.5 ± 0.3 µmol/L) [26]. Using an alpha of 0.05 and a power of 0.90, a sample size of 8 rats per group was calculated using nQuery (version 8.5.1, Boston, MA, USA). Rats that dropped out during the experiment were replaced. All data are expressed as mean ± standard deviation or median [interquartile range] and were analyzed using RStudio (version 4.3.2; Boston, MA, USA). Graphs were made using RStudio or GraphPad Prism 9.0 (GraphPad Software, La Jolla, CA, USA). Normality of distribution was tested with Q-Q plots and the Shapiro-Wilk test. Differences between the groups at termination were determined with an independent t-test or a Mann-Whitney U test. Mixed-effects models were used to test time-dependent differences between the groups. Repeated measures correlations were used to assess correlations. A *p*-value < 0.05 was considered statistically significant.

## Results

### Hemodynamics and blood gas analysis

**Fig. 2:**
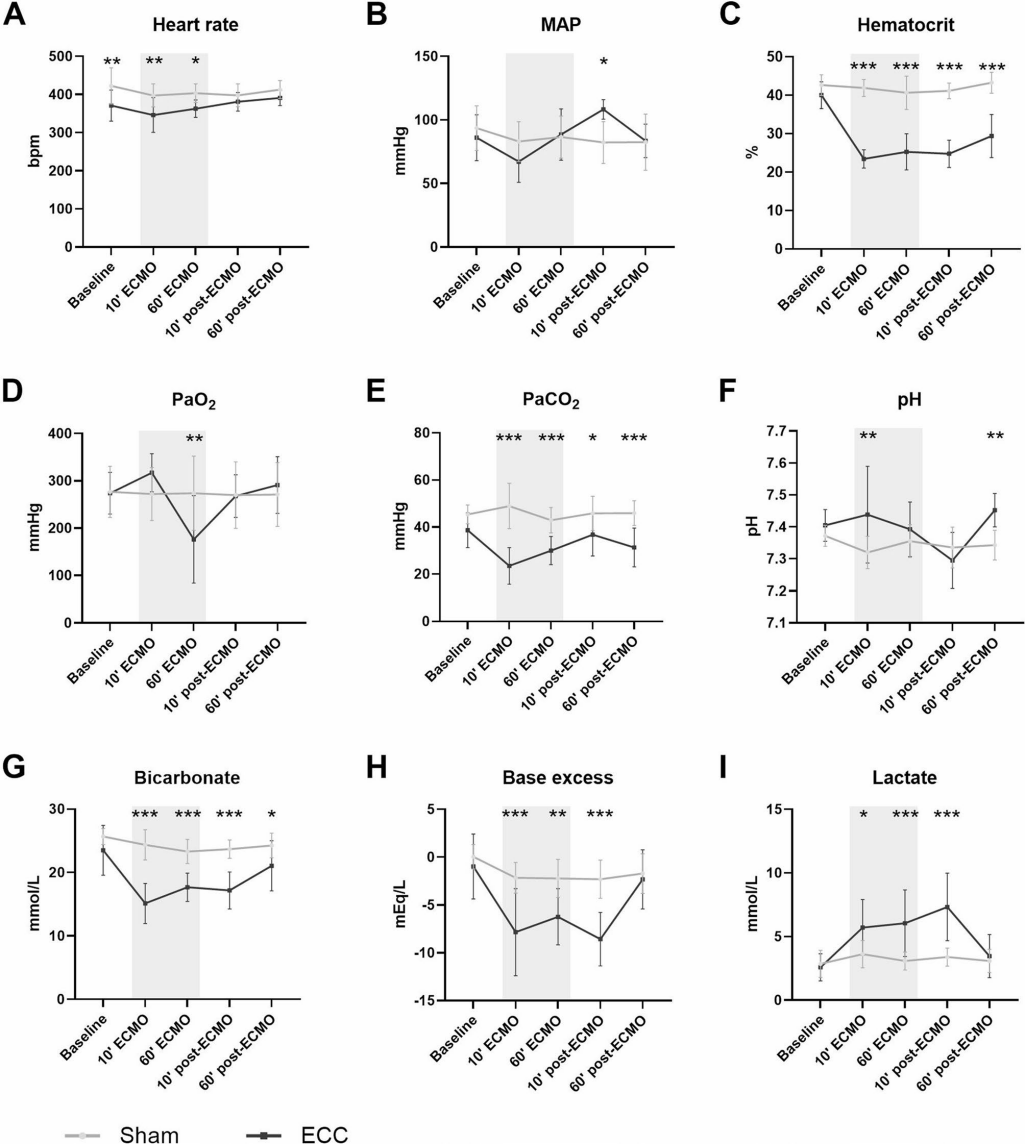
Hemodynamic parameters and arterial blood gas analyses. Heart rate (**A**), mean arterial pressure (MAP; **B**), hematocrit (**C**), partial oxygen pressure (PaO2; **D**), partial carbon dioxide pressure (PaCO2; **E**), pH (**F**), bicarbonate (**G**), base excess (**H**), and lactate (**I**) measured in rats prior to, during, and after extracorporeal membrane oxygenation (ECMO; black line, *n* = 8), or sham rats (grey line, *n* = 8). Data are presented as mean ± standard deviation. The grey box indicates the time on ECMO. * *p* < 0.05, ** *p* < 0.01, *** *p* < 0.001 between groups

ECMO initiation decreased heart rate (346 vs. 397 bpm, *p* = 0.0023), partial carbon dioxide pressure (23.5 vs. 48.9 mmHg, *p* < 0.001), bicarbonate (15.1 vs. 24.4 mmol/L, *p* < 0.001), and base excess levels (−7.9 vs. −2.2 mEq/L, *p* < 0.001) when compared to the sham group (Fig. 2). Furthermore, ECMO dropped hematocrit levels due to the priming fluid of the circuit (22.5 vs. 39.0%, *p* < 0.001; Fig. 2C), whereas levels remained stable in the sham group. Additionally, initiation of ECMO increased lactate levels (5.71 vs. 3.62 mmol/L, *p* = 0.014; Fig. 2I) compared to the sham group. One hour after weaning of ECMO, hemodynamic and blood gas parameters were restored to baseline levels, except for hematocrit which remained decreased (39.0 vs. 29.4%, *p* < 0.001). Furthermore, hematocrit (29.4 vs. 43.2%, *p* < 0.001), partial carbon dioxide pressure (31.3 vs. 45.9 mmHg, *p* < 0.001) and bicarbonate (21.1 vs. 24.3 mmol/L, *p* = 0.020) were lower whereas pH was higher (7.45 vs. 7.34, *p* = 0.005) in the ECMO group compared to the controls one hour after weaning from ECMO.

### ECMO increased intravascular hemolysis

**Fig. 3:**
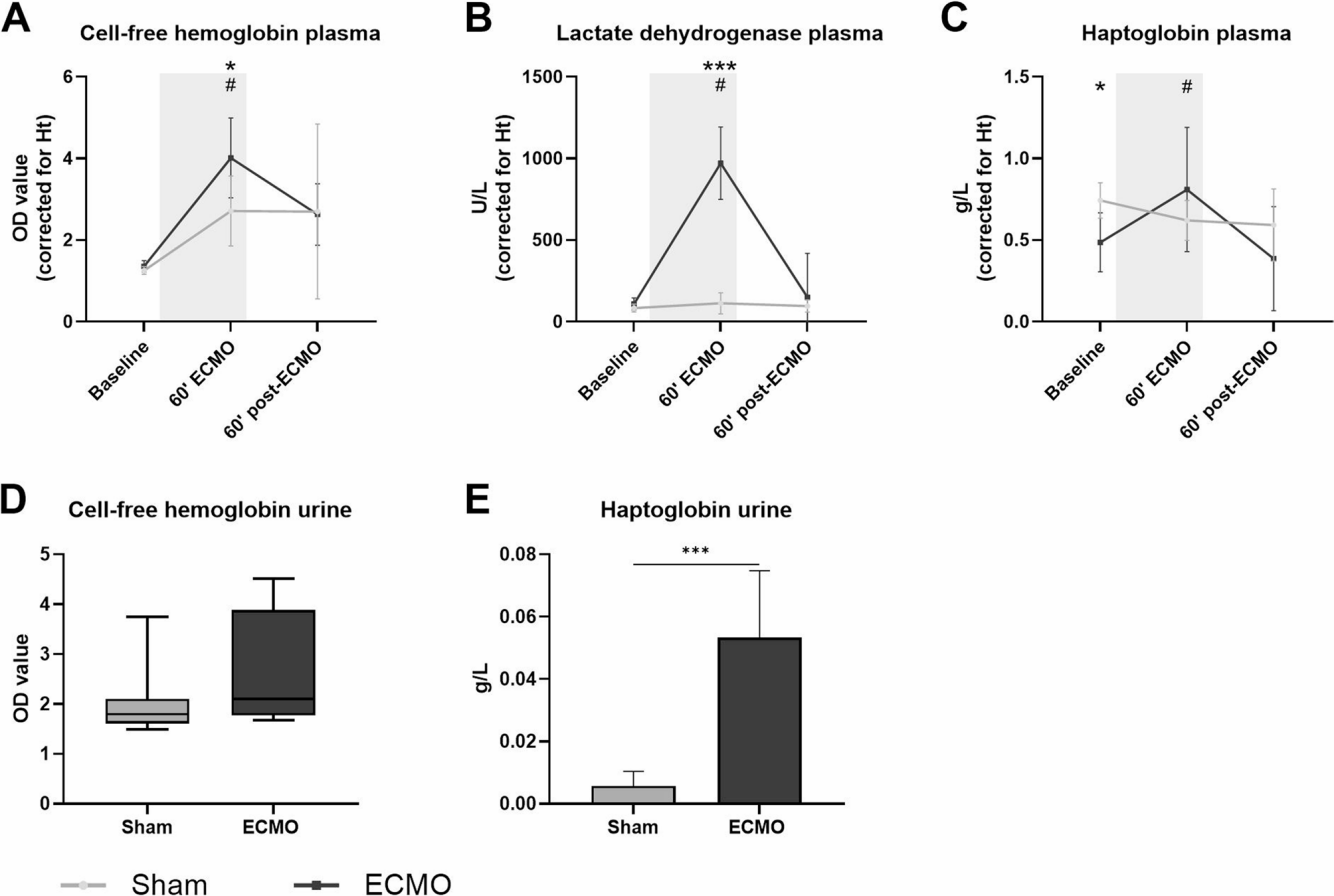
Circulating markers of hemolysis in plasma and urine. Circulating levels of cell-free hemoglobin (**A**), haptoglobin (**B**), and lactate dehydrogenase (**C**) in plasma measured in rats prior to, during, and after extracorporeal membrane oxygenation (ECMO; black line, *n* = 8), or sham rats (grey line, *n* = 8). Cell-free hemoglobin (**D**) and haptoglobin (**E**) were measured in urine samples collected after termination. Concentrations in plasma were corrected according to hematocrit levels. Data are presented as mean ± standard deviation or median with full range. The grey box in panel **A**-**C** indicates the time on ECMO. * *p* < 0.05, ** *p* < 0.01, *** *p* < 0.001 between groups, # *p* < 0.05 within the ECMO group compared to baseline

Initiation of ECMO increased circulating CFHb (4.01 vs. 1.36 OD, *p* < 0.001; Fig. 3A) and LDH (970.9 vs. 108.7 U/L, *p* < 0.001; Fig. 3B) compared to baseline. CFHb and LDH restored to baseline levels 60 min after weaning from ECMO. Baseline levels of haptoglobin differed between the groups, however both remained within the clinical reference range. After discontinuation of ECMO, haptoglobin levels decreased (0.81 vs. 0.44 g/L, *p* = 0.0015; Fig. 3C), whereas levels remained stable in the sham group during the experiment. Additionally, haptoglobin levels (0.053 vs. 0.0057 g/L, *p* < 0.001; Fig. 3E), but not CFHb levels (Fig. 3D), were higher in urine samples from rats supported by ECMO compared to the sham group.

### ECMO induced systemic inflammation and endothelial damage

**Fig. 4:**
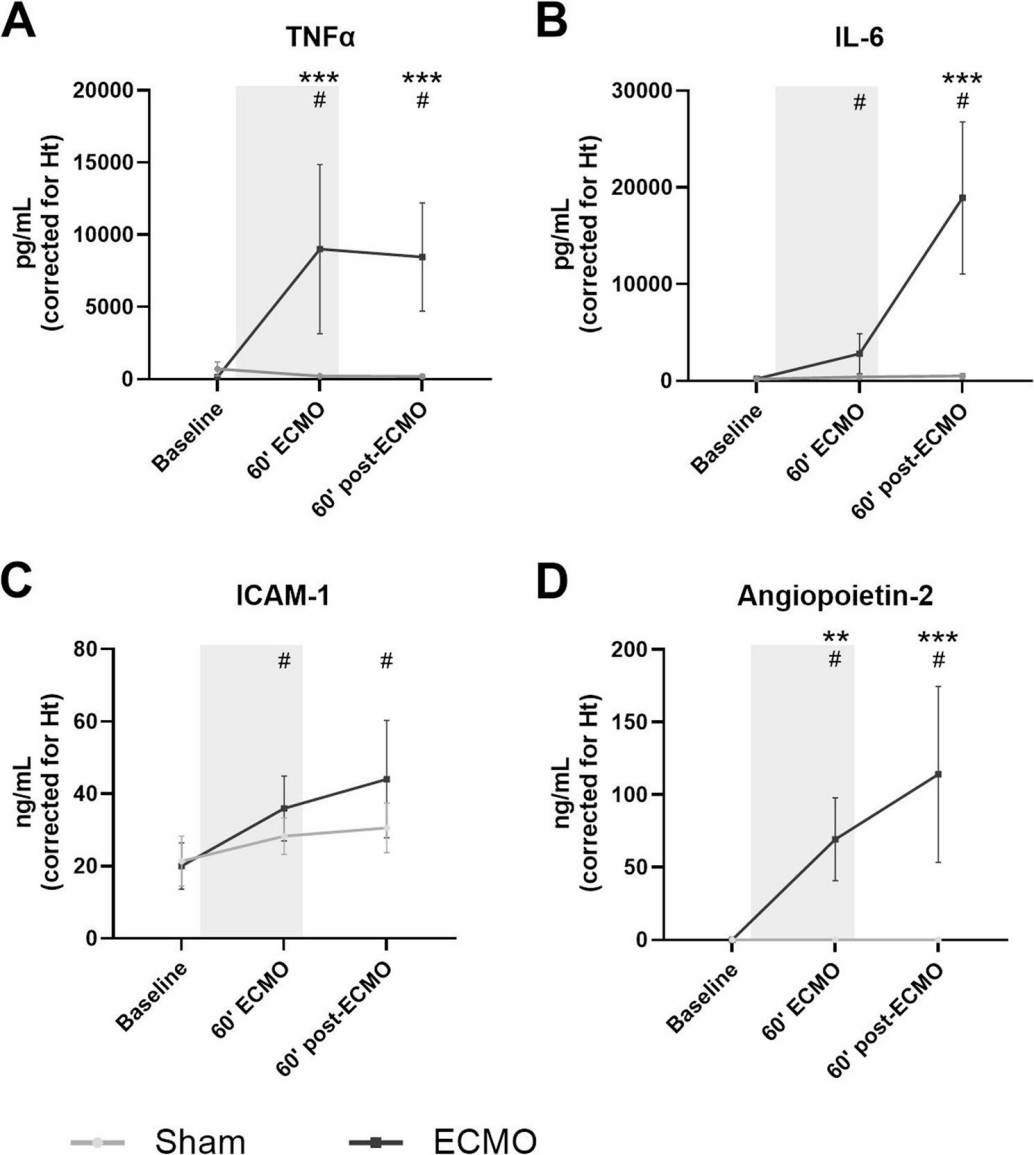
Circulating markers of inflammation and endothelial activation and damage. Circulating levels of tumor necrosis factor α (TNFα; **A**), interleukin-6 (IL-6; **B**), intercellular adhesion molecule 1 (ICAM-1; **C**), and angiopoietin-2 (**D**) measured in rats prior to, during, and after extracorporeal membrane oxygenation (ECMO; black line, *n* = 8), or sham rats (grey line, *n* = 8). Concentrations were corrected according to hematocrit levels. Data are presented as mean ± standard deviation. The grey box indicates the time on ECMO. * *p* < 0.05, ** *p* < 0.01, *** *p* < 0.001 between groups, # *p* < 0.05 within the ECMO group compared to baseline

ECMO increased circulating levels of TNFα (145 vs. 9002 pg/mL, *p* < 0.001; Fig. 4A), IL-6 (211 vs. 2809 pg/mL, *p* = 0.043; Fig. 4B), ICAM-1 (20.0 vs. 35.9 ng/mL, *p* < 0.001; Fig. 4C), and angiopoietin-2 (0 vs. 60.1 ng/mL, *p* < 0.001; Fig. 4D), whereas circulating levels remained stable in the sham group. Circulating levels of angiopoietin-2 kept rising despite discontinuation of ECMO (60.1 vs. 114.0 ng/mL; *p* < 0.001; Fig. 4D). Furthermore, levels of TNFα (8454 vs. 186 pg/mL, *p* < 0.001; Fig. 4A) and angiopoietin-2 (114.0 vs. 0, *p* < 0.001; Fig. 4D) concentrations were higher in the animals after 60 min of ECMO when compared to sham animals.

### ECMO disturbed microcirculatory perfusion

**Fig. 5:**
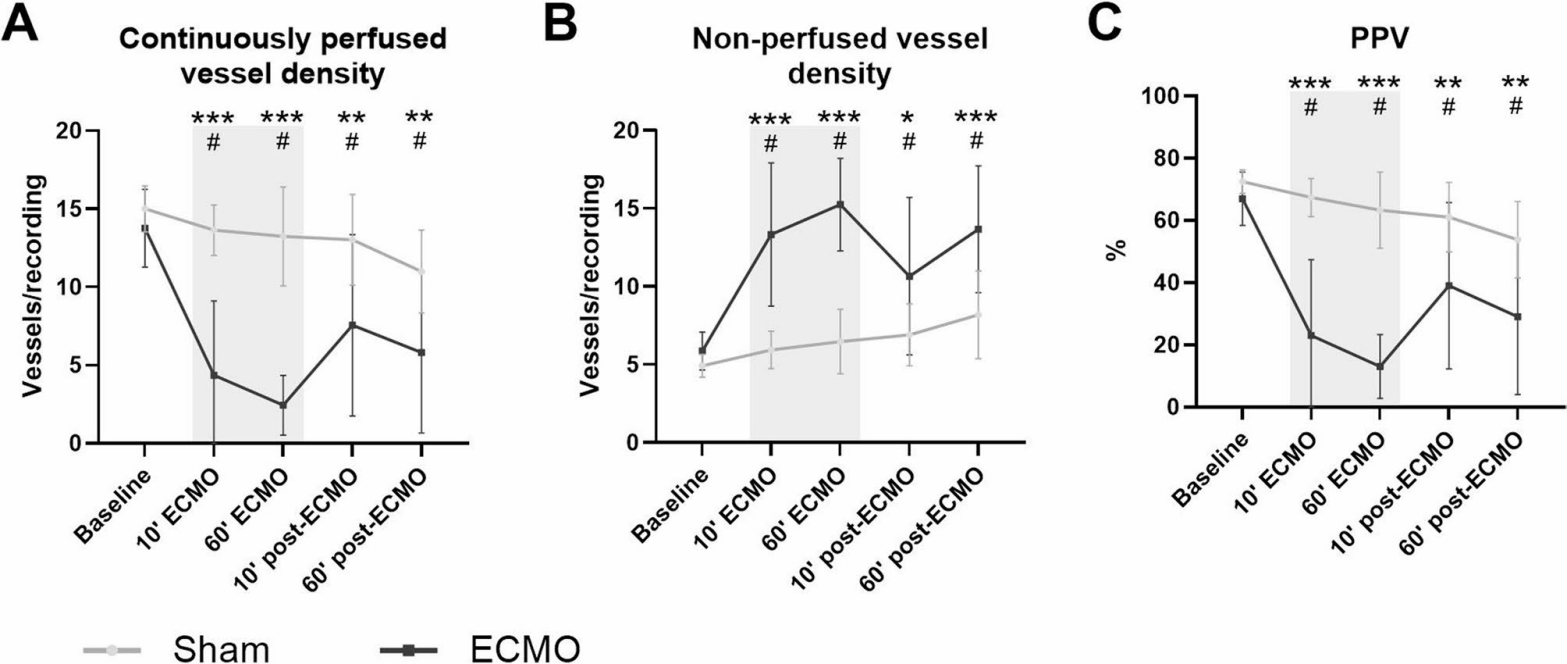
Microcirculatory perfusion of the cremaster muscle. Continuously perfused vessel density (**A**), non-perfused vessel density (**B**), and the proportion of perfused vessels (PPV; **C**) measured in the cremaster muscle of rats prior to, during, and after extracorporeal membrane oxygenation (ECMO; black line, *n* = 8), or sham rats (grey line, *n* = 8). Concentrations in plasma were corrected according to hematocrit levels. Data are presented as mean ± standard deviation. The grey box indicates the time on ECMO. * *p* < 0.05, ** *p* < 0.01, *** *p* < 0.001 between groups, # *p* < 0.05 within the ECMO group compared to baseline

The PVD (13.77 vs. 4.36 vessels/recording, *p* < 0.001; Fig. 5A) and the PPV (67.0 vs. 23.0%, *p* < 0.001; Fig. 5C) decreased after initiation of ECMO. During ECMO, PVD and PPV were continuously lower when compared to controls. Alongside this, the number of non-perfused vessels was higher in rats undergoing ECMO compared to sham (15.24 vs. 6.46 vessels/recording, *p* < 0.001; Fig. 5B). In addition, rats undergoing sham surgery showed a slight decrease in PPV over time (Fig. 5C). After discontinuation of ECMO, microcirculatory perfusion partially recovered but did not return to baseline levels again (29.1 vs. 67.0%, *p* < 0.001; Fig. 5C).

### ECMO resulted in renal injury

**Fig. 6:**
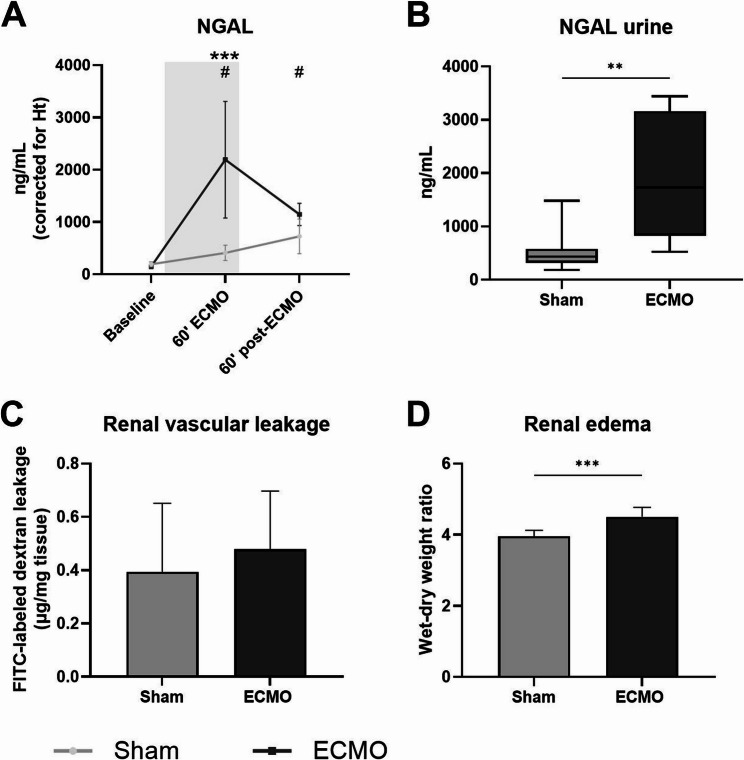
Kidney function and injury. Levels of neutrophil gelatinase-associated lipocalin (NGAL) measured in plasma (**A**) in rats prior to, during, and after extracorporeal membrane oxygenation (ECMO; black line, *n* = 8), or sham rats (grey line, *n* = 8). NGAL concentration in urine (**B**), renal FITC-labeled dextran extravasation (**C**) and wet-dry weight ratios (**D**) were determined after termination. Concentrations in plasma were corrected according to hematocrit levels. Data are presented as mean ± standard deviation. The grey box in panel A indicates the time on ECMO. * = *p* < 0.05 between groups, # *p* < 0.05, ** *p* < 0.01, *** *p* < 0.001 within the ECMO group compared to baseline

After 60 min of ECMO, circulating levels of NGAL in plasma, a marker of renal injury, significantly increased compared to baseline (2191 vs. 149 ng/mL, *p* < 0.001; Fig. 6A). Furthermore, urinary NGAL levels at termination were higher in the animals undergoing ECMO (1733 vs. 437 ng/mL, *p* = 0.006; Fig. 6B). Renal vascular leakage measured by FITC-labeled dextran extravasation was not different between the groups (Fig. 6C). However, wet-to-dry weight ratio showed increased renal edema in the group undergoing ECMO (4.50 vs. 3.96, *p* < 0.001; Fig. 6D).

### Cell-free hemoglobin is associated with microcirculatory perfusion disturbances

**Fig. 7:**
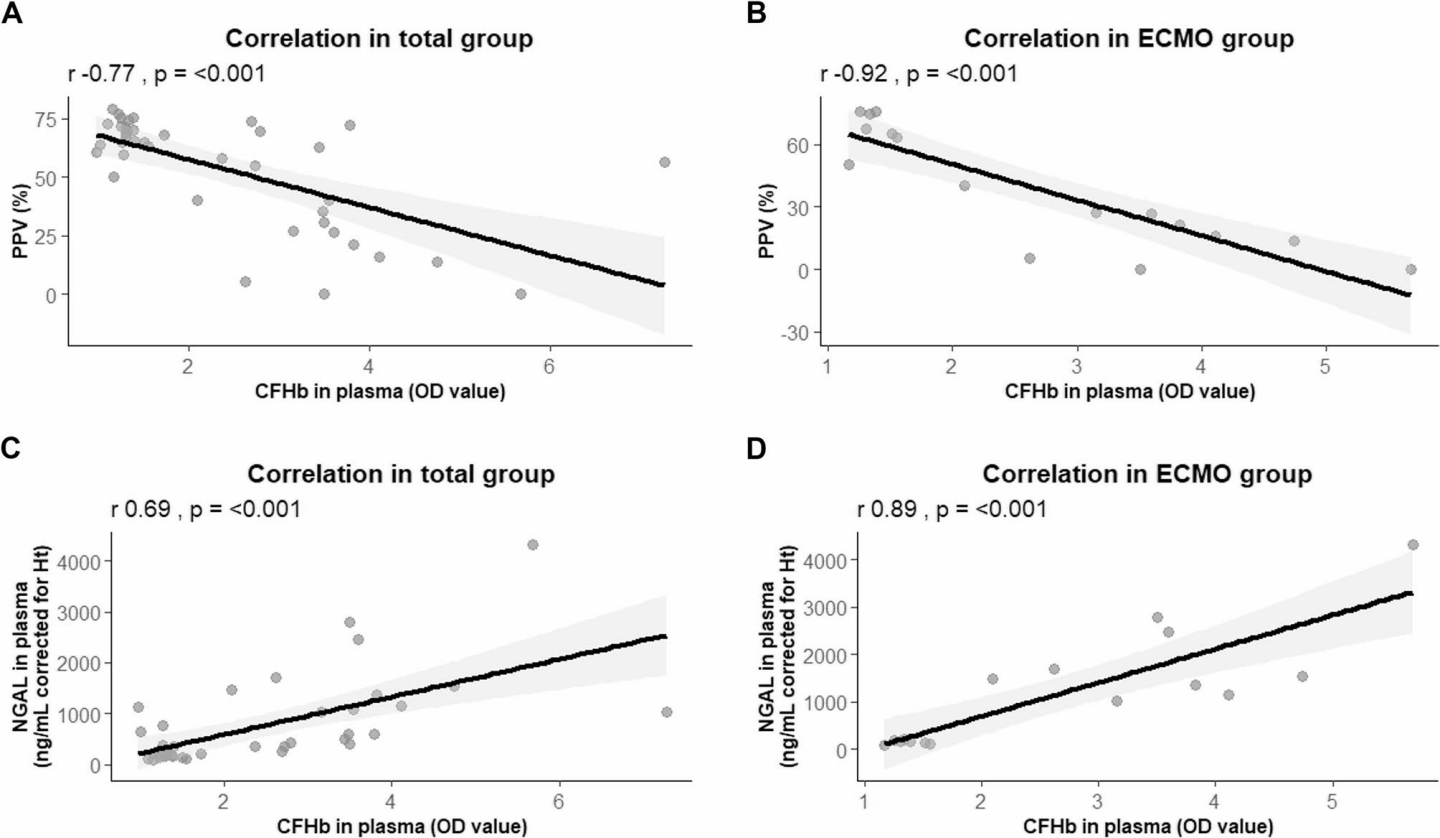
Repeated measures correlations between CFHb in plasma and PPV. Repeated measures correlations between cell-free hemoglobin (CFHb) in plasma and the proportion of perfused vessels (PPV) in the total group (**A**), CFHb in plasma and the PPV only the rats undergoing extracorporeal membrane oxygenation (ECMO; **B**), CFHb and neutrophil gelatinase-associated lipocalin (NGAL) in plasma in the total group (**C**), and CFHb and NGAL in plasma only in the rats undergoing ECMO (**D**). Data represent the repeated measures correlation with the 95% confidence interval (grey)

Repeated measures correlation showed a negative correlation between CFHb in plasma and PPV across both groups, with higher CFHb levels in plasma correlating with a lower PPV (*r*=−0.77, *p* < 0.001; Fig. 7A). This correlation was even stronger when only considering rats on ECMO (*r*=−0.92, *p* < 0.001; Fig. 7B). Linear mixed-effects model also showed that higher CFHb in plasma was associated with a lower PPV (β=−7.312, *p* = 0.0023). Furthermore, repeated measures correlation showed that rising levels of CFHb in plasma were strongly correlated with increased plasma levels of NGAL (*r* = 0.69, *p* < 0.001; Fig. 7C and *r* = 0.895, *p* < 0.001; Fig. 7D).

## Discussion

ECMO can be lifesaving, however, it is also associated with complications including hemolysis and AKI. Nonetheless, the pathophysiological mechanisms underlying AKI during ECMO are not yet fully understood. In this study, we aimed to elucidate the relationship between ECMO-induced hemolysis, microcirculatory perfusion disturbances and AKI in a rat model. We demonstrate that ECMO in rats induces inflammation, endothelial activation and endothelial damage. Moreover, ECMO in rats leads to a significant rise in CFHb and LDH, which represent intravascular hemolysis. This was paralleled by microcirculatory perfusion disturbances, renal edema and kidney injury. Interestingly, increased levels of CFHb in rats on ECMO were strongly correlated with decreased microcirculatory perfusion and elevated markers of kidney injury. These findings support the hypothesis that ECMO-induced hemolysis contributes to endothelial damage and microcirculatory perfusion disturbances, thereby exacerbating kidney injury.

The Extracorporeal Life Support Organization (ELSO) guidelines recognize hemolysis as an underrecognized complication of extracorporeal circulation [4], with CFHb being associated with AKI and worse patient outcome [5, 6, 27]. We demonstrate an increase in circulating CFHb and LDH levels upon ECMO initiation in our rat model. These findings align with studies reported in a systematic review showing that CFHb levels increase after the start of ECMO in patients [28]. Although CFHb levels returned to baseline after ECMO discontinuation in our rat model, haptoglobin levels decreased, indicating ongoing scavenging of CFHb. Similarly, retrospective studies in ECMO patients have reported a depletion of haptoglobin during ECMO [29, 30]. These studies also show that this depletion is associated with adverse outcomes and increased mortality in patients on VV-ECMO [29, 30]. Overall, these findings support the clinical relevance of our ECMO rat model in replicating hemolysis-associated pathophysiology.

Microcirculatory perfusion is crucial for the delivery of oxygen and nutrients to tissues, and disturbances are associated with organ failure in critically ill patients [31, 32]. Microcirculatory perfusion has been extensively investigated in patients undergoing cardiac surgery with cardiopulmonary bypass, however, data in patients supported with ECMO is scarce [33]. We observed a significant reduction in microcirculatory perfusion following ECMO initiation in rats. Despite partial recovery after ECMO discontinuation, microcirculatory perfusion did not return to baseline, indicating prolonged microvascular dysfunction. In contrast, macrocirculatory parameters did return to baseline levels and mean arterial pressure remained above 50 mmHg, suggesting a loss of hemodynamic coherence. These findings are consistent with previous studies in which ECMO was shown to impair microcirculatory perfusion in rats [14–17]. Importantly, we observed a strong negative correlation between CFHb levels and microcirculatory perfusion, especially in rats exposed to ECMO. This correlation is strongly supported by a prior study by Hanssen and colleagues who have shown that the administration of CFHb in rats results in impaired intestinal microcirculatory perfusion [34]. Furthermore, a study in septic patients shows that increased plasma CFHb levels after a red blood cell transfusion are associated with impairment of the microcirculatory perfusion [35]. Taken together, these results support the hypothesis that CFHb directly affects microcirculatory perfusion.

Additionally, ECMO led to kidney injury, as represented by an increase in NGAL in plasma and urine, along with the development of kidney edema. These results represent the clinical situation where AKI affects almost two-thirds of patients on ECMO support [3]. Since NGAL is an early indicator of tubular damage, the increased NGAL levels observed in our rats suggest the presence of tubular injury. This renal damage may also explain the presence of haptoglobin in the urine, which typically reflects increased glomerular permeability or impaired tubular reabsorption due to injury. Notably, we observed a strong correlation between CFHb and circulating NGAL. Since the kidneys are the primary route for clearance of CFHb when scavenging systems are depleted, they are highly susceptible to damage during hemolysis [11, 36]. Our results are consistent with previous studies indicating that CFHb induces tubular toxicity [10, 11]. Nonetheless, the exact mechanisms underlying CFHb-induced toxicity are not fully elucidated. Our proposed pathway involved may be through endothelial activation, leading to increased vascular permeability and kidney edema. The observed association between CFHb and microcirculatory perfusion in our study supports this hypothesis, highlighting a possible role for endothelial damage in CFHb-mediated kidney injury [18, 19, 37]. Previously, we have shown that pharmacological protection of the endothelium reduced tissue edema and improved microcirculatory perfusion in rats undergoing ECMO, highlighting the important role of the endothelium in critical illness [14, 16, 17]. The increase in angiopoietin-2, a marker of endothelial damage and a key regulator of endothelial barrier function, in our study suggests a shift towards an activated, pro-inflammatory endothelial phenotype. Notably, angiopoietin-2 levels continued to rise even after discontinuation of ECMO, highlighting the persistence of endothelial damage.

Alongside these findings, we observed a decrease in partial carbon dioxide pressure during ECMO. Since mechanical ventilation was reduced during ECMO, gas exchange primarily occurred across the oxygenator membrane which could have led to excessive CO_2_ removal. Additionally, the concurrent reduction in bicarbonate could reflect both dilutional effects from circuit priming and a buffering response to rising lactate levels. Interestingly, although pH remained within the normal range, this may have resulted from a mixed acid-base disturbance with elements of both respiratory alkalosis and metabolic acidosis. The rise in lactate during ECMO further supports the notion of impaired tissue perfusion or reduced oxygen delivery. This aligns with our microcirculatory findings and may also reflect decreased oxygen-carrying capacity due to hemodilution and hemolysis. These findings further highlight the complex interplay between hemolysis, endothelial damage, and microcirculatory perfusion disturbances during ECMO. Further mechanistic studies are needed to establish a causal link and explore potential therapeutic strategies to mitigate CFHb-induced endothelial damage and kidney dysfunction.

### Strengths and limitations

This study has several limitations that should be considered. Firstly, our rat model involves a relatively short period of extracorporeal circulation, whereas ECMO support in patients can last for days to weeks. However, due to the higher metabolic rate in rats, physiological changes occur more rapidly than in humans. Moreover, we have previously demonstrated the clinical relevance of our model, as similar endothelial and microcirculatory disturbances have been observed in our rat model and patients undergoing cardiopulmonary bypass [14, 16, 38, 39]. Secondly, we conducted this study in healthy animals, whereas patients requiring ECMO have severe underlying conditions. While this may limit direct clinical translatability, it focusses on the adverse effects of extracorporeal circulation itself, without the confounding influence of the underlying disease. Thirdly, clinical ECMO systems have coatings and centrifugal pumps instead of roller pumps which may result in less hemolysis compared to our model. However, clinical studies observe ECMO-induced hemolysis during ECMO support, and the current model makes it possible to investigate the pathophysiological pathways involved in hemolysis-induced morbidity during ECMO support. Finally, our findings demonstrate a strong association between CFHb levels and microvascular perfusion. Though, causality cannot be stated based on these results. Future studies using targeted interventions to modulate CFHb levels or endothelial function are needed to further elucidate the pathways underlying ECMO-induced hemolysis, endothelial damage and kidney injury.

## Conclusion

In conclusion, we demonstrate that ECMO induces hemolysis, endothelial damage, microcirculatory perfusion disturbances, and kidney injury in rats. Our findings suggest that CFHb plays an important role in the pathophysiology of AKI, possibly via endothelial activation and damage resulting in microcirculatory perfusion disturbances. These results have important clinical implications, as microvascular dysfunction and kidney injury are major complications in patients on ECMO support. Monitoring CFHb levels and assessing markers of endothelial activation and damage could help identify patients at risk for AKI. Future studies should elucidate the causal relationship between CFHb and endothelial damage and explore whether interventions targeting CFHb can improve microcirculatory perfusion and preserve kidney function during ECMO.

## Data Availability

The datasets used and/or analyzed during the current study are available from the corresponding author on reasonable request.

## Abbreviations

AKI: Acute kidney injury
ARRIVE: Animal Research: Reporting of In Vivo Experiments
CFHb: Cell-free hemoglobin
ECMO: Extracorporeal membrane oxygenation
ELISA: Enzyme linked immunosorbent assay
ELSO: Extracorporeal life support organization
ICAM-1: Intercellular adhesion molecule 1
IL-6: Interleukin-6
LDH: Lactate dehydrogenase
MAP: Mean arterial pressure
NGAL: Neutrophil gelatinase-associated lipocalin
PEEP: Positive end-expiratory pressure
PPV: Proportion of perfused vessels
PVD: Continuously perfused vessel density
TNFα: Tumor necrosis factor α
VV-ECMO: Veno-venous extracorporeal membrane oxygenation
